# Metadherin: A Therapeutic Target in Multiple Cancers

**DOI:** 10.3389/fonc.2019.00349

**Published:** 2019-05-03

**Authors:** Gourav Dhiman, Neha Srivastava, Mehendi Goyal, Emad Rakha, Jennifer Lothion-Roy, Nigel P. Mongan, Regina R. Miftakhova, Svetlana F. Khaiboullina, Albert A. Rizvanov, Manoj Baranwal

**Affiliations:** ^1^Department of Biotechnology, Thapar Institute of Engineering and Technology, Patiala, India; ^2^Faculty of Medicine and Health Sciences, School of Medicine, University of Nottingham, Nottingham, United Kingdom; ^3^Faculty of Medicine and Health Sciences, School of Veterinary Medicine and Science, University of Nottingham, Nottingham, United Kingdom; ^4^Institute of Fundamental Medicine and Biology, Kazan Federal University, Kazan, Russia; ^5^Department of Microbiology and Immunology, University of Nevada, Reno, NV, United States

**Keywords:** metadherin, chemoresistance, microRNA, immunotherapy, cancer

## Abstract

Altered expression of many genes and proteins is essential for cancer development and progression. Recently, the affected expression of metadherin (MTDH), also known as AEG-1 (Astrocyte Elevated Gene 1) and Lyric, has been implicated in various aspects of cancer progression and metastasis. Elevated expression of MTDH/AEG-1 has been reported in many cancers including breast, prostate, liver, and esophageal cancers, whereas its expression is low or absent in non-malignant tissues. These expression studies suggest that MTDH may represent a potential tumor associated antigen. MTDH also regulates multiple signaling pathways including PI3K/Akt, NF-κB, Wnt/β-catenin, and MAPK which cooperate to promote the tumorigenic and metastatic potential of transformed cells. Several microRNA have also been found to be associated with the increased MTDH expression in different cancers. Increased MTDH levels were linked to the tumor chemoresistance making it an attractive novel therapeutic target. In this review, we summarize data on MTDH function in various cancers.

## Introduction

Advances in cancer prevention and early diagnosis significantly improved the cancer treatment outcomes. Still, cancers remain one of the most challenging global healthcare. Approximately 18 million new cancer cases and 9.6 million malignancy related deaths were expected in 2018 (1). One of the major challenges in cancer therapy is its specificity, where only cancers become affected without injuring the healthy cells. Therefore, advances in identification of tumor antigens are essential for targeting exclusively tumor cells. Tumor antigens are classified as tumor specific, tumor associated and cancer testis antigens (2). Tumor associated antigens (TAA) are over-expressed in tumors and these can be used as immunotherapeutic targets. In the effort of identification of this targets, 15 Astrocyte Elevated Genes have been characterized, of which metadherin (MTDH) was shown to be the most relevant to tumorigenesis. MTDH, also known as Astrocyte Elevated Gene-1(AEG-1) or LYRIC (Lysine Rich CEACAM1), is a putative TAA (3–5). MTDH was identified by *in vivo* phage display screening as a protein which was responsible for the breast to lung cancer metastasis possessing extracellular domain called lung homing domain (4). Further investigations revealed MTDH expression in various cancer types capable of metastasis (3–5).

**Figure 1:**
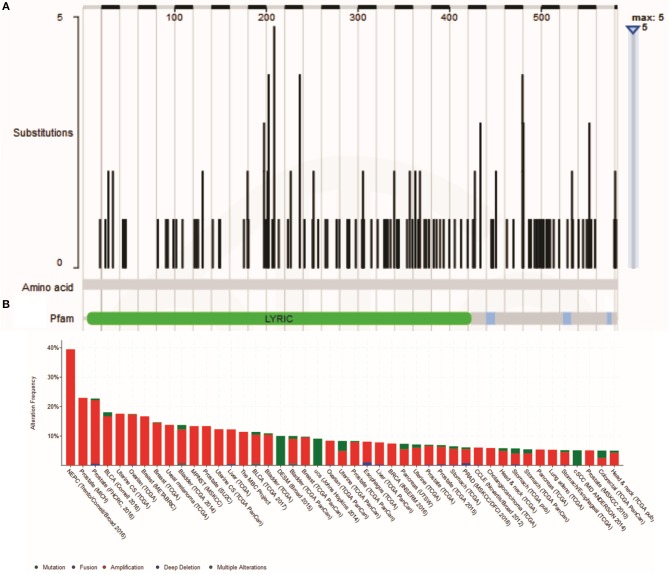
**(A)** Cancer-related Metadherin mutations (https://cancer.sanger.ac.uk/cosmic) **(B)** Metadherin mutations in many cancer types. The results was generated by the TCGA Research Network (http://www.cbioportal.org/).

**Figure 2:**
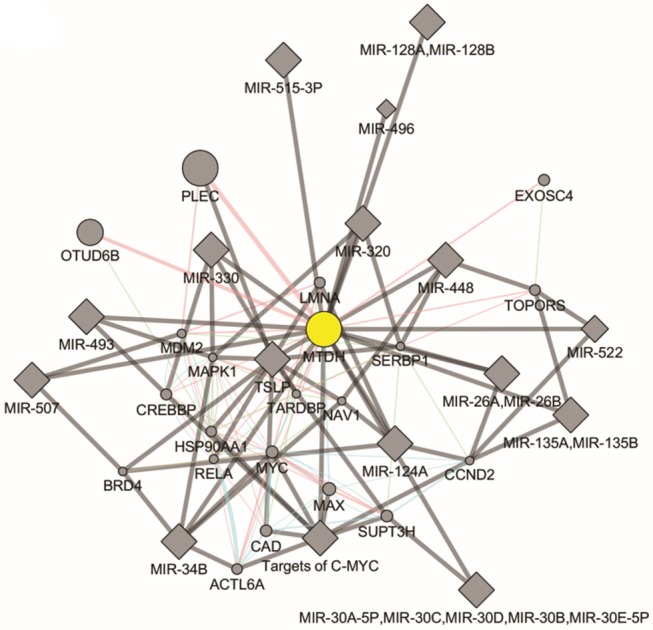
Molecular pathways of Metadherin generated by GeneMANIA Cytoscape plugin (https://genemania.org/).

There are two MTDH isoforms coded on chromosome eight. Amplification of 8q22, including the MTDH locus is associated with chemoresistance and metastasis in aggressive breast cancer (6). Multiple cancer associated mutations in the MTDH gene (Figure 1A) are reported in the COSMIC database (7). Furthermore, altered expression, copy number and mutations of MTDH (Figure 1B) have been identified in many cancer types as reported in the cBIO database (8). There is increasing evidence in functional interactions of MTDH and important pro-oncogenic pathways, including MYC-mediated processes (Figure 2). It appears that the main role of MTDH is associated with tumor chemoresistance and metastasis (6). Here we present an overview of the MTDH expression and function in various cancers as well as its potential as an intrinsic treatment modulating agent.

## MTDH Regulation and Molecular Functions

MTDH is a type-two transmembrane protein containing an extracellular lung homing domain which is implicated in breast cancer metastasis to the lung (4). MTDH encodes a single-pass transmembrane protein with the molecular mass of 64-kDa expressed mainly in the endoplasmic reticulum and perinuclear space (5). In polarized epithelial cells, it colocalizes with tight junction protein ZO-1 and occludin (3); however MTDH is not a native component of tight junctions but become incorporated during tight junction complex maturation. The sub-cellular location of MTDH protein varies depending upon physiological state of the cell (9). In non-malignant tissue, MTDH was shown expressed in the nucleus, whereas in malignant cells it becomes translocated into the cytoplasm (10). It is believed that cytoplasmic translocation of MTDH promotes disease progression by mediating mechanisms that support pro-angiogenesic and metastatic pathways.

**Figure 3:**
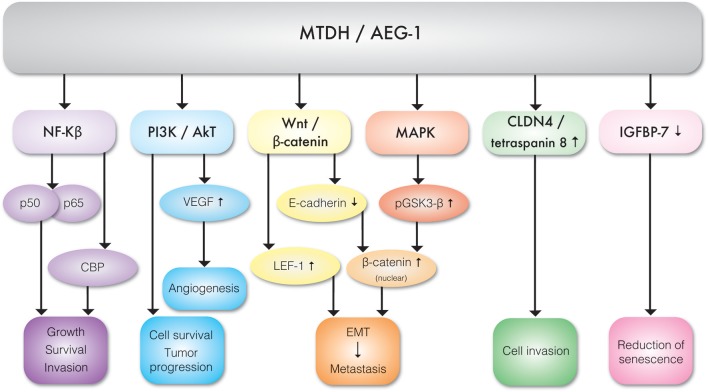
Molecular interactions between MTDH/AEG-1 and various effector molecules of signal transduction pathways exhibiting different biological functions.

It appears that TNF-α is the key regulator of MTDH expression. TNF-α upregulates MTDH expression via NF-κB pathways. TNF-α causes NF-κB nuclear translocation and consequent interaction with MTDH, which is essential for activation of downstream genes (11). The N-terminal domain of MTDH interacts with NF-κB and triggers gene expression via several convergent mechanisms (9). NF-κB nuclear translocation coincides with a significant reduction of IκBα level, suggesting MTDH involvement in IκBα degradation. Studies have also revealed that MTDH interacts with Cyclic AMP-responsive element binding protein–binding protein (CBP) which is a NF-κB coactivator (9, 12). Hence, MTDH may function as a bridging element among p50–p65, NF-κB CBP, and the basal transcription machinery and therefore consequent induction of NF-κB related gene expression enhances migration and invasion (9) (Figure 3). MTDH promoted NF-κB gene expression results in anchorage independent cell growth (10), possibly mediated by direct activation of matrix metalloproteinase 1 (MMP1) expression (13). MTDH also serves as a link between NF-κB and matrix metalloprotease 9 (MMP9) expression (14, 15).The role of MTDH/AEG-1 as an endoplasmic reticulum (ER)-associated cytoplasmic RNA binding protein has been recently reported by Meng et al. (16) where MTDH/AEG-1 was also found in complex with other RNA binding proteins. More recently Hsu et al. (17) identified the MTDH/AEG-1: RNA interactome using unbiased genome wide methods including HITS-CLIP (high-throughput sequencing of RNA isolated by crosslinking immunoprecipitation) and PAR-CLIP (photoactivatable ribonucleoside-enhanced crosslinking and immunoprecipitation) which revealed that the MTDH/AEG-1 RNA interactome includes the organelle protein-encoding transcripts as well as secretory and cytosolic protein-encoding mRNAs (17).

## MTDH Expression and Function in Cancer

MTDH is involved in multiple cancer associated cellular signaling pathways, most notably in the context of this review, pro-angiogenesis and pro-metastasis pathways (Figure 3, Table 1). MTDH/AEG-1 promotes cell survival, inhibits apoptosis, and promotes tumor progression via multiple PI3K/Akt pathways (18). Emdad et al. (19) further showed that MTDH/AEG-1 promotes an invasive phenotype and angiogenesis via PI3K-Akt. The potential role of MTDH in angiogenesis is related to its functional link to vascular endothelial growth factor (VEGF), IGFBP7, and epithelial–mesenchymal transition (EMT) markers. It was shown that MTDH increases VEGF expression via the PI3K/Akt pathway in head and neck squamous cell (20). MTDH is also associated with expression of the E-cadherin, β-catenin, snail, and N-cadherin epithelial-mesenchymal transition (EMT) markers in hepatocellular carcinoma cells (21). There is also evidence of a functional link between MTDH and pro-survival mechanisms mediated by the lymphoid enhancer binding factor 1 (LEF-1) and GSK3β components of the Wnt/β-catenin pathway in chronic lymphocytic leukemia (22) and hepatocellular carcinoma (23). Elevated expression of MTDH in hepatocellular carcinoma results in the up-regulation of multiple genes and signaling pathways such as the activation of ERK 42/44 and p38 Mitogen Activated Protein Kinase (MAPK) signaling pathway (24). Activation of ERK 42/44 and p38 MAPK enhanced GSK3β phosphorylation which in turn drives β-catenin nuclear translocation and thereby activate Wnt signaling (24).

**Table 1:** MTDH containing gene ontologies.

**GENE ONTOLOGIES**
**Molecular Functions**
GO:0000988:transcription factor activity, protein binding
GO:0000989:transcription factor activity, transcription factor binding
GO:0001085:RNA polymerase II transcription factor binding
GO:0003712:transcription cofactor activity
GO:0003713:transcription coactivator activity
GO:0003723:RNA binding
GO:0003725:double-stranded RNA binding
GO:0008134:transcription factor binding
GO:0044822:poly(A) RNA binding
GO:0051059:NF-kappaB binding
**Cellular Components**
GO:0005635:nuclear envelope
GO:0005923:bicellular tight junction
GO:0016324:apical plasma membrane
GO:0016604:nuclear body
GO:0031965:nuclear membrane
GO:0043296:apical junction complex
GO:0045177:apical part of cell
GO:0046581:intercellular canaliculus
GO:0048471:perinuclear region of cytoplasm
GO:0070160:occluding junction
**Molecular Functions**
GO:0007043:cell-cell junction assembly
GO:0010508:positive regulation of autophagy
GO:0031663:lipopolysaccharide-mediated signaling pathway
GO:0043297:apical junction assembly
GO:0045766:positive regulation of angiogenesis
GO:0051092:positive regulation of NF-kappaB transcription factor activity
GO:0051896:regulation of protein kinase B signaling
GO:0051897:positive regulation of protein kinase B signaling
GO:0070830:bicellular tight junction assembly
GO:1904018:positive regulation of vasculature development

Also, MTDH/AEG-1 can up regulate Claudin 4 (CLDN4) and tetraspanin which enhance cell invasion (23). Chen et al. have shown that the increased expression of MTDH/AEG-1 frequently overserved in hepatocellular carcinoma downregulates expression of the IGFB7 tumor suppressor and thereby contributes to cancer progression (25). It has also been shown that the knockdown of MTDH causes increased sensitivity to Panobinostat (LBH589) and tumor necrosis factor-a-related apoptosis-inducing ligand (TRAIL) combination treatment. As TRAIL can trigger apoptosis in cancer cells and LBH589 augments the sensitivity of cancer cells to TRAIL induced apoptosis. It can be inferred that over expression of MTDH may negatively influence apoptosis and cell cycle checkpoints and thereby promote cell survival (26).

## Clinical Significance of MTDH Expression in Cancer

MTDH was shown to be expressed in ductal carcinoma *in situ* (DCIS) of the breast (27). Increased MTDH expression is also implicated in prostate cancer (10, 28). Also MTDH functionally interacts with the Ha-Ras oncogene and leads to tumor development and progression in melanocytes (5). Expression of MTDH is implicated in breast cancer stem cell (CSC) growth and tumor resistance to paclitaxel and trastuzumab (29–32). Elevated expression of MTDH was also reported in salivary gland tumors and is associated with poorer outcomes (33). Increased expression of MTDH is also implicated in hepatocellular carcinoma recurrence and metastasis which remains one of five most commonly diagnosed cancers worldwide, largely attributable to chronic viral hepatitis (HBV, HCV), and alcoholism (20, 34–36). Increased MTDH expression is also associated with mechanisms of metastasis in colorectal cancer (CRC) (15, 37) including activation of MMP9. Increased MTDH is also associated with AKT/PI3K mediated mechanisms of metastasis in head and neck squamous cell carcinoma (HNSCC) patients (38). The significance of MTDH regulation of cancer growth was confirmed using miRNA-375 and MTDH knockdown experiments in HNSCC model (35). MTDH can regulate the cancer cell metastasis by actin cytoskeletal remodeling in gastric and non-small cell lung cancer (39, 40). Accordingly, the downregulation of MTDH expression could induce remodeling of the actin cytoskeleton and inhibit epithelial-mesenchymal transition in gastric cancer cell lines (MKN45 and AGS) (39).

## Role of Increased MTDH Expression in Treatment Resistance

Many mechanisms of anticancer drug resistance have been described in cancer cells and have been reviewed elsewhere recently (41, 42). There is a strong evidence linking MTDH expression to the resistance to multiple cancer therapeutics, including tamoxifen, trastuzumab, and paclitaxel (6, 16, 30, 31, 43, 44).Chemo-resistance in MTDH expressing cells was shown associated with activation of autophagy. Autophagy related mechanisms were shown to protect tumor cells from metabolic stress caused by the anticancer drugs cisplatin and paclitaxel (45). Experimental suppression of MTDH lead to an increased sensitivity to doxorubicin in cancer cells (46–48). These MTDH targeting therapeutic approaches could applied not only to treat cancer, but also to prevent, reverse or delay the chemo resistance.

## Micro RNAs Regulation of MTDH Expression

**Figure 4:**
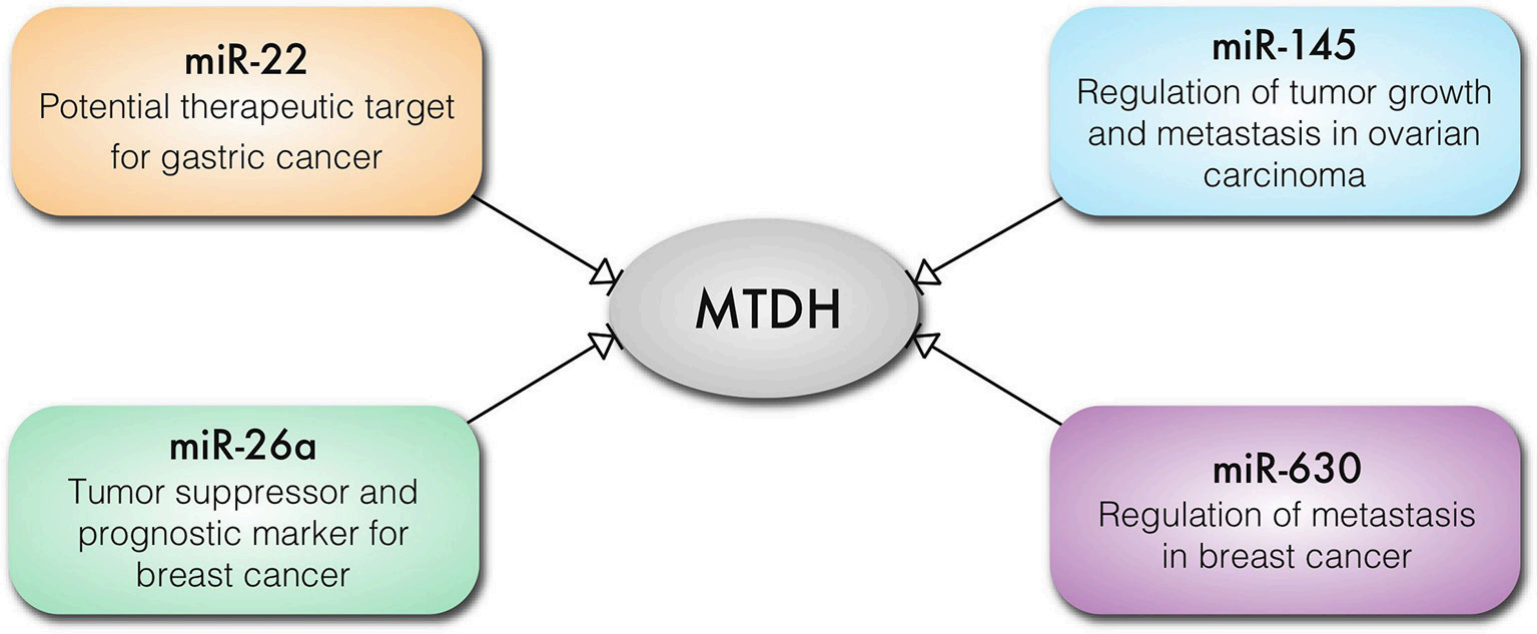
Key cancer related miRNAs that regulate the expression of MTDH in different types of cancer cells.

It is well-established that micro-RNAs (miRs) play important, if subtle, roles in the regulation of gene expression and translation in cellular differentiation and proliferation. Unsurprisingly, aberrant expression of oncogenic miRs (onco-miRs) and tumor suppressor miRs is implicated in cancer pathogenesis (49, 50). MTDH regulates expression of miRs and in turn is also regulated by miRs (Figure 4). MTDH knockdown inhibits angiogenetic properties of the MDA-MB-231 breast cancer cell line, which is mediated by downregulation of the oncomir *miR-21* (51). In contrast, reduction in *miR-630* expression results in increased MTDH expression in breast cancer (52) (Figure 4). Expression of *miR-145* is lost in high-grade serous ovarian carcinoma resulting in an increased expression of MTDH (53) (Figure 4). Loss of activity of the p53 tumor suppressor results in decreased or loss of *miR-145* expression, contributing to an increased MTDH levels (53). Similarly, *miR-342-3p* functions as a tumor suppressor by targeting MTDH in human osteosarcoma (54) and prostate cancer (55). Expression of *miR-26a* regulates MTDH levels, where the loss of *miR-26a* in triple negative breast cancer cells (TNBC) leads to increased MTDH and acts as a prognostic marker for breast cancer outcome (56) (Figure 4). Similarly, upregulation of MTDH in gastric cancer may occur as a consequence of reduced *miR-22* expression. Moreover, *miR-22* can prevent gastric cancer cell proliferation and invasion, suggesting its potential therapeutic efficacy (57) (Figure 4). Thus, it can be concluded that miRNAs are crucially involved in regulation of MTDH expression in malignancies.

## MTDH as a Potential Target for Cancer Therapeutics

MTDH plays an important role in progression and metastasis of many cancers. Although the understanding of the MTDH cancer promoting mechanisms is incomplete, there is a compelling evidence that MTDH regulates multiple signaling pathways which cooperate to promote the tumorigenic and metastatic potential of transformed cells. Additionally there is new evidence suggesting that functional inhibition of MTDH function could be the novel approach to treat cancer. For example, MTDH in breast cancer down regulates expression of the PTEN (Phosphatase and tensin homolog) tumor suppressor via NF-κB mediated pathways and contributes to the HER2-targeting therapy resistance (31). Thus, MTDH may represent an interesting therapeutic target for treatment of HER2+ breast cancers. For example, Bortezomib, is an FDA-approved proteasome inhibitor used in multiple myeloma treatment and acts (in part) by reducing MTDH activity (58). Studies have further demonstrated that RNAi-mediated knockdown of MTDH reduces angiogenesis by down regulating the ERK1/2 signaling (51).

Immunotherapy targeting the tumor antigens has been an area of intense research. Expression of MTDH is increased in many different cancer types. Importantly, auto-antibodies against the MTDH protein have been detected in cancer patients confirming its immunogenicity and supports MDTH as a potential immunotherapy target (59). We recently identified three T-cell epitopes within the MTDH protein supporting its potential value as a cancer vaccine target (60). Consistent with this a DNA vaccine designed to induce an anti-MTDH CD8^+^ mediated immune response inhibited cancer cell proliferation and lung metastases in a mouse breast cancer xenograft model (47). Given that MTDH/AEG-1 expression is increased in many cancer types and proof of principle experiments support it as an effective immunotherapy target, further research is now warranted to test such treatments efficacy in advanced preclinical cancer models.

## Conclusion

MTDH is pro-oncogenic factor playing multifaceted and diverse roles in cancer progression. Its association and central role in regulating signaling pathways such a MAPK, wnt/β-catenin, PI3K/AkT, NF-κβ pathways in various cancers shows that it plays a vital role in metastasis. MTDH contribution to chemo and radiotherapy resistance provides a new direction for the development of anticancer therapeutics. Multiple mechanisms converge to promote expression of MTDH in cancers. Further studies are therefore warranted to determine whether the elevated MTDH expression has prognostic value for development of the malignancy. Given its increased expression in many cancer types it would be important to determine whether MTDH represents a feasible target for cancer therapy, including immune-therapy. For these reasons there is now an urgent need to determine the clinical and therapeutic significance of increased MTDH expression in cancer.

## Author Contributions

GD originated the idea for writing the review and wrote about MTDH association in different cancers. NS and MG wrote the following sections: micro RNAs regulation of MTDH expression and MTDH target for cancer therapy. ER, JL-R, and NM were involved in writing the MTDH regulation and molecular functions and preparing the original figures. RM and SK contributed to the clinical significance of MTDH expression in cancer. AR managed the different collaborations during the writing of the review and contributed to editing the manuscript. MB was overall responsible for coordinating and managing multisite collaboration, and writing the manuscript.

### Conflict of Interest Statement

The authors declare that the research was conducted in the absence of any commercial or financial relationships that could be construed as a potential conflict of interest.
